# Socioeconomic Status and Campylobacteriosis, Connecticut, USA, 1999–2009

**DOI:** 10.3201/eid2007.131333

**Published:** 2014-07

**Authors:** Kelley Bemis, Ruthanne Marcus, James L. Hadler

**Affiliations:** Yale School of Public Health Connecticut Emerging Infections Program, New Haven, Connecticut, USA (K. Bemis, R. Marcus, J.L. Hadler)

**Keywords:** Campylobacter, GIS, socioeconomic status, poverty, campylobacteriosis, Connecticut, bacteria, *Suggested citation for this article*: Bemis K, Marcus R, Hadler JL.Socioeconomic status and campylobacteriosis, Connecticut, USA, 1999–2009 [letter].Emerg Infect Dis [Internet]. 2014 Jul [*date cited*]. http://dx.doi.org/10.3201/eid2007.131333

**To the Editor:** *Campylobacter* is the second most common bacterial cause of foodborne gastrointestinal illnesses in the United States and the leading cause of these illnesses in Connecticut (*1*). It is also the leading identifiable cause of Guillain-Barré syndrome in the United States and all industrialized countries in which it has been studied (*2*). According to the Foodborne Disease Active Surveillance Network (FoodNet), campylobacteriosis incidence in the United States is increasing (*1*). Clarification of the epidemiology of campylobacteriosis is needed to control and prevent infection.

Socioeconomic status (SES) measures have not been explored in the United States as determinants for *Campylobacter* infection. Although individual SES measures are not routinely collected in FoodNet, street address of patient residence is. Following the recommended method of the Public Health Disparities Geocoding Project (*3*), we used census tract–level poverty as an SES measure for analysis. We attempted to geocode patient residences for all campylobacteriosis cases reported in Connecticut during 1999–2009 and to categorize them into 4 groups on the basis of percentage of residents in the census tract living below the federal poverty line: 0–<5%, 5%–<10%, 10%–<20%, and >20%. The average annual age-adjusted (on the basis of 2000 US Census data for Connecticut) incidence rate was calculated for each of 4 census tract-level neighborhood SES (i.e., neighborhood poverty) categories for all years combined and for 3 periods (1999–2002, 2003–2005, and 2006–2009). In addition, age group-specific rates were calculated for case-patients in the 4 SES categories. We used the χ^2^ test for trend to assess the statistical significance of observed gradients of incidence across SES levels.

We geocoded 5,708 (95.9%) of the 5,950 campylobacteriosis cases reported during 1999–2009 to census tract level. The average annual crude incidence rate was 15.9 per 100,000 population; average age-specific incidence ranged from 9.4 in the 10–19-year age group to 18.1 in the >50-year age group. We found a strong dose-response relationship between higher campylobacteriosis incidence and higher neighborhood SES. Average annual age-adjusted incidence was 10.1 (95% CI 9.1–11.1) for the lowest SES group (>20% below poverty), 11.9 (95% CI 11.0–12.9) for the 10%–<20% group, 14.8 (95% CI 14.0–15.7) for the 5%–<10% group, and 16.9 per 100,000 (95% CI 16.3–17.4) for the highest SES group (0–<5% below poverty) (p<0.001 byχ^2^for trend). A strong SES gradient was also consistent and significant (p<0.001 by χ^2^ for trend) for each of the 3 periods.

Incidence within age groups by neighborhood SES level is shown in the Figure. For all age groups >10 years, incidence of campylobacteriosis increased as neighborhood SES increased (p<0.001 for each category by χ^2^ for trend). However, for children 0–<10 years of age, the socioeconomic gradient seen in teenagers and adults reversed direction; incidence increased as neighborhood SES decreased (p<0.001 by χ^2^ for trend). Because only 51% of case reports included information on race and ethnicity, we were unable to examine whether SES gradients occurred within each major racial/ethnic group in Connecticut.

**Figure:**
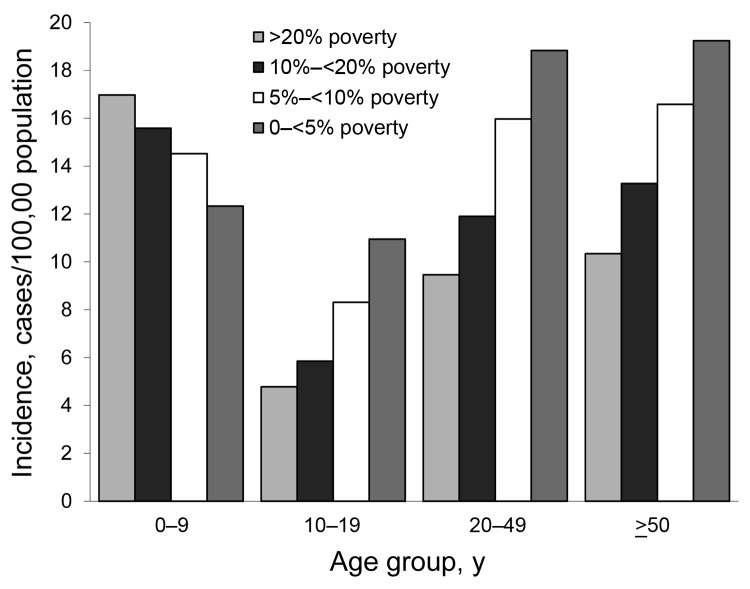
Average annual incidence rates for *Campylobacter* infection, by age group and neighborhood poverty level, Connecticut, 1999 −2009. Census tract groupings were determined by percentage of residents living below the federal poverty level on the basis of data from the 2000 US Census.

Previous studies using similar area-based methods in Denmark; Manitoba, Canada; Queensland, Australia; and Scotland also found an association between *Campylobacter* infection incidence and higher area-based SES (*4**–**7*). A true higher prevalence of major campylobacteriosis risk factors among patients with a higher SES might explain these findings, but these results could also indicate surveillance artifacts if persons at higher SES levels are more likely to seek health care and have an organism-specific diagnosis made. We believe the former hypothesis is more likely for several reasons. First, major risk factors for adult campylobacteriosis at FoodNet sites are international travel and eating out at restaurants (*8*). We examined these factors in Connecticut by using the 3 FoodNet population surveys (*9*) that occurred during the study period (2000–2001, 2002–2003, and 2006–2007) and found that these factors were associated with higher SES (K. Bemis, unpub. data). Second, we found that higher incidence in children <10 years of age was associated with lower SES, a finding that would not be expected if children living in poorer neighborhoods were less likely to receive a diagnosis of campylobacteriosis. Last, we examined Connecticut-specific data from the same 3 FoodNet population surveys (*9*) and found that high-income adults who had diarrhea were no more likely than those with lower incomes to visit a healthcare provider and have a stool specimen taken (K Bemis, unpub. data). The finding that children living in poorer census tracts were at higher risk than those in higher SES areas could conceivably reflect a higher rate of exposure to *Campylobacter* spp. in the home. However, this hypothesis needs verification. In addition, studies in other parts of the United States are needed to corroborate this study’s findings.

We conclude that *Campylobacter* control efforts, at least in Connecticut, should take into consideration the groups with highest age-specific, SES-related incidence. Area-based SES measures should be more widely used when analyzing surveillance data.
